# Andrographolide modulates glucose metabolism in visceral adipose tissue in an Alzheimer's disease obese mouse model

**DOI:** 10.1016/j.jbc.2025.110607

**Published:** 2025-08-16

**Authors:** Paulina Ormazabal, Camila Gherardelli, Cristina Pinto, Evrim Servili, Carolina Mendez-Orellana, G. William Wong, Roberto Contreras-Díaz, Pedro Cisternas, Nibaldo C. Inestrosa

**Affiliations:** 1 Escuela de Obstetricia, Facultad de Ciencias para el Cuidado de la Salud, Universidad San Sebastián, Santiago, Chile; 2 Departamento de Biología Celular y Molecular, Facultad de Ciencias Biológicas, Pontificia Universidad Católica de Chile, Santiago, Chile; 3 Centro de Excelencia en Biomedicina de Magallanes (CEBIMA), Escuela de Medicina Universidad de Magallanes, Punta Arenas, Chile; 4 Programa de Biología Celular y Molecular, Instituto de Ciencias Biomédicas, Facultad de Medicina, Universidad de Chile, Santiago, Chile; 5 Health Sciences Department, Faculty of Medicine, Pontificia Universidad Católica de Chile, Santiago, Chile; 6 Región Metropolitana & Radiology Department, Erasmus MC- University Medical Center Rotterdam the Netherlands, Rotterdam, the Netherlands; 7 Department of Physiology, The Johns Hopkins University School of Medicine, Baltimore, Maryland, USA; 8 Center for Metabolism and Obesity Research, The Johns Hopkins University School of Medicine, Baltimore, Maryland, USA; 9 Centro Regional de Investigación y Desarrollo Sustentable de Atacama (CRIDESAT), Universidad de Atacama, Copiapó, Chile; 10 Núcleo de investigación en nutrición y ciencias alimentarias (NINCAL), Facultad de Salud y Ciencias sociales, Universidad de las Américas, Santiago, Chile

**Keywords:** Alzheimer's disease, andrographolide, obesity, visceral adipose tissue, glucose metabolism

## Abstract

Midlife obesity and high adiposity are recognized as risk factors for Alzheimer's disease (AD), with visceral adipose tissue (VAT) playing a central role due to its endocrine and metabolic activity. Disturbances in VAT metabolism and adipokine secretion exacerbate AD pathology. Andrographolide (Andro), known for its anti-diabetic properties, enhances neuronal glucose uptake and alleviates AD pathology. However, its effects on VAT metabolism in AD remain unexplored. This study aimed to investigate the impact of Andro on glucose metabolism in VAT using a high-fat diet (HFD)-induced obesity model in AD mice (APP/PS1). APP/PS1 mice were fed an HFD and received Andro injections (2 mg/kg, three times a week for 16 weeks). VAT samples were analyzed for glucose uptake, glycolytic rate, pentose phosphate flux, ADP-ATP levels, gene expression, and enzymatic activity of glucose metabolic regulators. In APP/PS1 mice, HFD significantly increased glucose uptake and reduced GLUT4 expression in VAT, effects counteracted by Andro (*p* < 0.05). Andro-treated HFD-fed mice exhibited reduced glucose oxidation through glycolysis (*p* < 0.05), leading to decreased ATP production (*p* < 0.05). Andro administration restored the activity of key glycolytic enzymes and mitigated several HFD-induced metabolic alterations (*p* < 0.05). The study reveals significant metabolic changes in the VAT of obese APP/PS1 mice and highlights Andro's potential as a therapeutic agent for addressing VAT impairment induced by obesity in AD.

Alzheimer's disease (AD) is the most prevalent senile dementia, marked by a gradual cognitive decline (1). AD is defined by extracellular amyloid-β (Aβ) deposits, known as amyloid plaques, and intracellular accumulations of hyperphosphorylated tau protein (2, 3). A reduction in glucose consumption in the brains of patients with AD is observed, even before the appearance of clinical symptoms, and is closely linked to disease progression (4, 5, 6, 7). The causes of reduced glucose metabolism remain unclear; however, multiple studies suggest that enhancing glucose uptake could potentially improve memory and cognitive function (8, 9, 10).

In recent years, metabolic disorders, such as obesity, diabetes, and hypertension, have emerged as significant risk factors for the development of dementia (11, 12). Among them, obesity has garnered significant attention due to its escalating prevalence, projected to affect around 60% of the global adult population by 2030 according to the World Health Organization (13, 14). Epidemiological studies have associated midlife obesity with an elevated risk of developing AD (15, 16, 17, 18). Indeed, the accumulation of body fat in the abdominal cavity, primarily composed of visceral adipose tissue (VAT), has been implicated in eliciting inflammatory responses, potentially contributing to the neuroinflammation observed in AD patients (19, 20, 21). Moreover, chronic low-grade inflammation that accompanies an increase in VAT mass has been shown to disrupt the endocrine function of the adipose tissue, resulting in impaired secretion of adipokines, molecules that have been involved in AD pathogenesis (15, 22, 23, 24, 25, 26). Interestingly, impaired VAT metabolism and the burden of cerebral Aβ are correlated (27). While the exact connection between obesity, VAT impairment, and AD remains elusive, evidence suggests that obesity-related insulin resistance could be a central contributor (28, 29). Intriguingly, peripheral insulin resistance and type 2 diabetes have been demonstrated to decrease Aβ clearance, reduce gray matter volume, increase tau deposition, and accelerate cognitive decline in individuals with AD (30). Therefore, pharmacological interventions aimed at restoring adipocyte function hold promise as therapeutic strategies to alleviate symptoms associated with AD.

Andrographolide (Andro), a labdane diterpene and the primary active component of *Andrographis paniculata*, exhibits anti-inflammatory, antioxidant, and antidiabetic properties both *in vivo* and *in vitro* (31, 32, 33, 34, 35). Importantly, prior studies conducted by our group and others have revealed Andro’s ability to modulate key metabolic players and restore glucose metabolism in various cell and animal models (36, 37, 38). However, it remains unclear whether Andro can also restore the obesity-associated metabolic changes in VAT during AD. Our findings collectively support the use of Andro as a compound able to restore metabolic alterations in the VAT of animals with obesity, positioning it as a potential pharmacological candidate for restoring obesity-induced VAT alterations in AD.

## Results

### Andro reduces glucose uptake in VAT of HFD-fed animals

**Figure 1:**
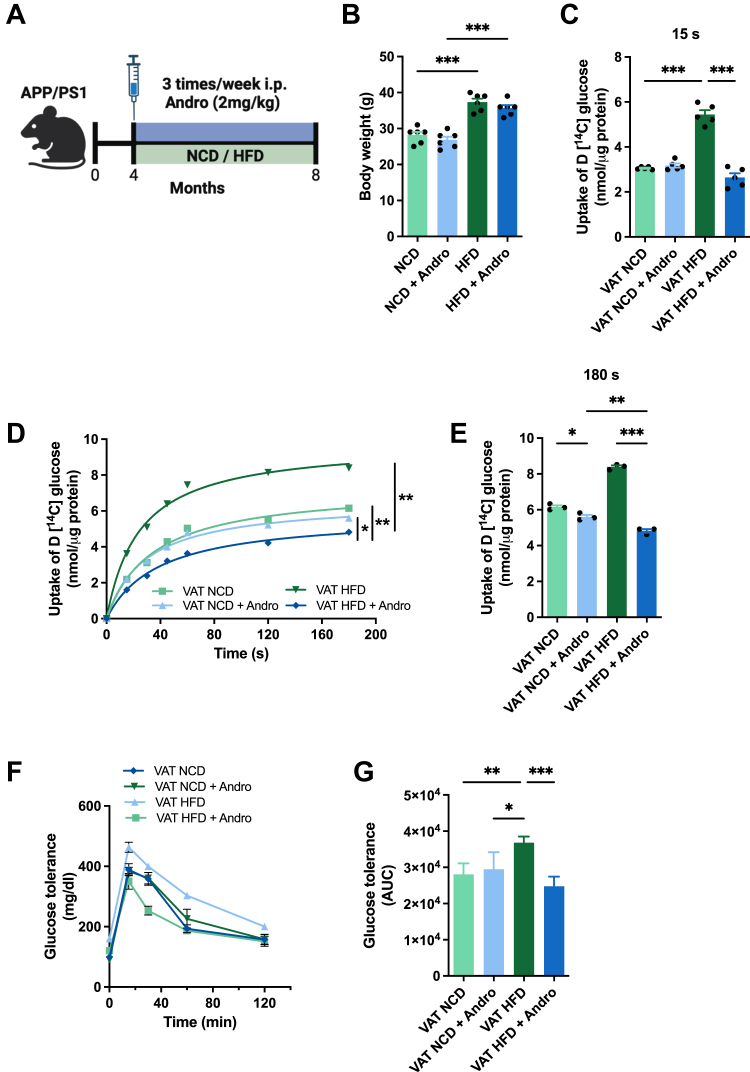
**Andro reduces glucose uptake in VAT of HFD-fed animals.***A*, schematic representation of experimental design. Four-month-old APP/PS1 mice were subjected to two dietary regimens: a normal chow diet (NCD) or a high-fat diet (HFD) and received 4 months of intraperitoneal Andro or saline injections until they were euthanized at 8 weeks of age. *B*, body weights of mice following indicated treatments. One-way ANOVA followed by Bonferroni's *post hoc* test was performed. *C*, uptake of D [^14^C] Glucose of VAT measured at 15 s. One-way ANOVA followed by Bonferroni's *post hoc* test was performed. *D*, D [^14^C] Glucose uptake kinetic curve of VAT obtained from NCD or HFD-fed animals, in the presence or absence of Andro, measured over 180 s. Two-way ANOVA followed by Bonferroni's *post hoc* test was performed. *E*, uptake of D [^14^C] Glucose at 180 s of D. One-way ANOVA followed by Bonferroni's *post hoc* test was performed. *F*, glucose tolerance measured over 120 min. *G*, graph depicting the area under the curve (AUC) of F. One-way ANOVA followed by Bonferroni's *post hoc* test was performed. Data is represented as the mean values ± SD (n = 3–6 mice).

To study the impact of Andro on glucose metabolism in VAT, we subjected four-month-old APP/PS1 animals to two dietary regimens: a normal chow diet (NCD) and a high-fat diet (HFD), both accompanied by 4 months of intraperitoneal Andro injections (Fig. 1*A*). As expected, HFD-fed animals exhibited a significant increase in body weights (Fig. 1*B*). To further characterize the systemic metabolic status of the animals, we evaluated key biochemical parameters in blood samples, including glucose, insulin, triglycerides, cholesterol, and liver enzymes. These data are presented in Table 1, highlighting the metabolic alterations induced by HFD, and showing that Andro treatment normalizes some of these changes. To assess whether Andro alters VAT's glucose metabolism, we measured glucose uptake (glucose-C^14^) in NCD and HFD-fed animals. Interestingly, HDF resulted in a strong increase in glucose uptake in VAT compared to NCD. In addition, while no differences were observed in the NCD group, Andro significantly reduced glucose uptake in the VAT of HFD-fed animals (Fig. 1*C*). Further analysis of VAT glucose uptake over 180 s revealed that HFD-fed animals exhibited approximately a 40% increase compared to NCD controls at 180 s (Fig. 1, *D* and *E*). Significantly, Andro administration effectively lowered VAT glucose uptake in HFD-fed animals, reaching similar levels observed in NCD animals. To evaluate the systemic impact of Andro on glucose homeostasis, we performed a glucose tolerance test (GTT) in all experimental groups (Fig. 1*F*). The area under the curve (AUC) analysis revealed a significant increase in glucose intolerance in HFD-fed animals compared to NCD controls. Importantly, Andro treatment significantly reduced the AUC in HFD-fed animals (Fig. 1*G*), indicating a partial improvement in glucose tolerance.

**Table 1:** Metabolic and liver function parameters in APP/PS1 mice under different dietary conditions

Blood parameters	NCD	NCD + Androndro	HFD	HFD + Andro
Glucose (mg/ml)	93.2 ± 9.2	91.3 ± 5.8	140 ± 20.7	121.7 ± 19.8
Cholesterol (mg/dl)	124.7 ± 7.7	121 ± 11.3	191.3 ± 15	150.2 ± 12.8 ∗∗∗
HOMA-IR	0.2 ± 0.02	0.2 ± 0.01	1.3 ± 0.2	0.8 ± 0.1 ∗∗∗
Triglycerides (mg/dl)	87.3 ± 7	86 ± 5.8	166.8 ± 21.3	139.6 ± 11.4 ∗
Insulin (mU/L)	0.7 ± 0.08	0.7 ± 0.05	3.7 ± 0.4	2.7 ± 0.3 ∗∗∗
Alkaline phosphatase (UI/L)	74.72 ± 16.4	92.9 ± 12.3	85.5 ± 13.5	84.1 ± 12.5
AST (UI/L)	93.3 ± 11.3	96.4 ± 9.4	92.5 ± 6.8	87.7 ± 8.1

Values are presented as mean ± SD for each experimental group: normal chow diet (NCD), NCD + andrographolide (Andro), high-fat diet (HFD), and HFD + Andro. Statistical significance was assessed by one-way ANOVA followed by Bonferroni’s *post hoc* test.

∗*p* < 0.05, ∗∗*p* < 0.01, ∗∗∗*p* < 0.001 vs. HFD group.

### HFD-induced glycolysis is reversed by Andro, promoting the PPP pathway

**Figure 2:**
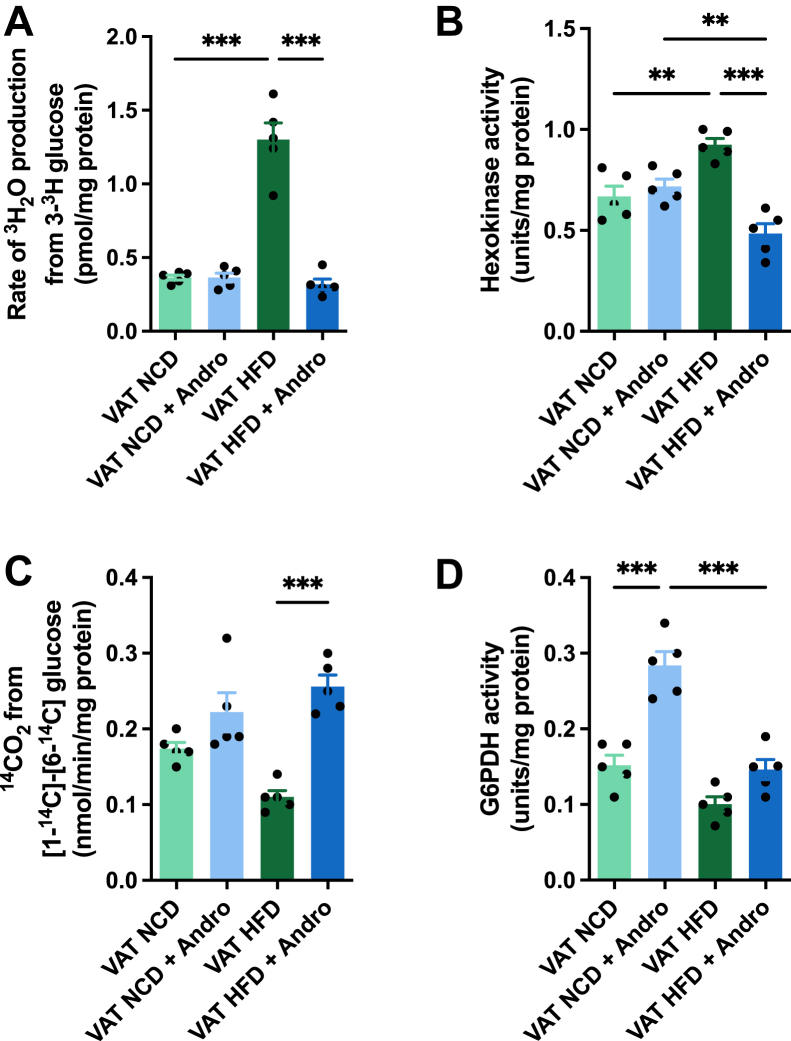
**HFD promotes glycolysis in VAT and Andro mitigates this effect.***A*, glycolytic rate, measured by ^3^H_2_O production, of VAT after indicated treatments. One-way ANOVA followed by Bonferroni's *post hoc* test was performed. *B*, hexokinase activity in VAT. One-way ANOVA followed by Bonferroni's *post hoc* test was performed. *C*, pentose phosphate flux of VAT, measured by the rate of ^14^CO_2_ released from [1-^14^C] and [6-^14^C]. One-way ANOVA followed by Bonferroni's *post hoc* test was performed. *D*, bar graph of the activity of glucose-6-phosphate dehydrogenase (G6PDH) in VAT. One-way ANOVA followed by Bonferroni's *post hoc* test was performed. Data are represented as the mean values ± SD (n = 5 mice).

Due to the observed effect on glucose metabolism and to further characterize the impact of HFD and Andro on VAT on glucose metabolic pathways, we also investigated the rate of glycolysis in VAT. Our findings revealed a 3.5-fold increase in the rate of glycolysis in the VAT of HFD-fed animals compared to those on the NCD (Fig. 2*A*). Notably, this effect was eliminated in animals that received Andro. Subsequently, we measured the activity of hexokinase (HK), an enzyme responsible for catalyzing the initial step in glycolysis. While HK activity showed no differences among the NCD groups, there was a significant increase in the VAT of HFD-fed animals (Fig. 2*B*). Interestingly, the Andro administration reduced HK activity within the HFD group to levels even lower than those observed in the VAT of the NCD group. Once inside the cell, glucose can also undergo oxidation *via* the pentose phosphate pathway (PPP). Thus, to measure PPP rates, we administered VAT with 1-^14^C and 6-^14^C glucose and followed the ^14^CO_2_ release. Our results showed no differences in the rate of glucose metabolized by PPP in NCD animals, independent of the presence of Andro (Fig. 2*C*). Conversely, HFD caused a modest, though statistically not significant, reduction in PPP rates compared to NCD groups. Interestingly, this effect was counteracted by the presence of Andro. Given the observed changes in PPP, and to validate our findings, we also measured the activity of glucose-6-phosphate dehydrogenase (G6PDH), a pivotal and rate-limiting enzyme in the PPP. Our results revealed an approximately 0.9-fold increase in G6PDH within the VAT of animals fed with NCD and treated with Andro in comparison to the control group (Fig. 2*D*). Although we noted a substantial reduction in G6PDH activity in the VAT of HFD-fed animals compared to NCD, the presence of Andro did not rescue this effect. Together, our results suggest that HFD enhances glucose utilization in VAT through glycolysis while decreasing PPP activity. Notably, Andro effectively reverses these effects, restoring the metabolic profile.

### Andro induces a decrease in ATP synthesis in the VAT of HFD-fed mice

**Figure 3:**
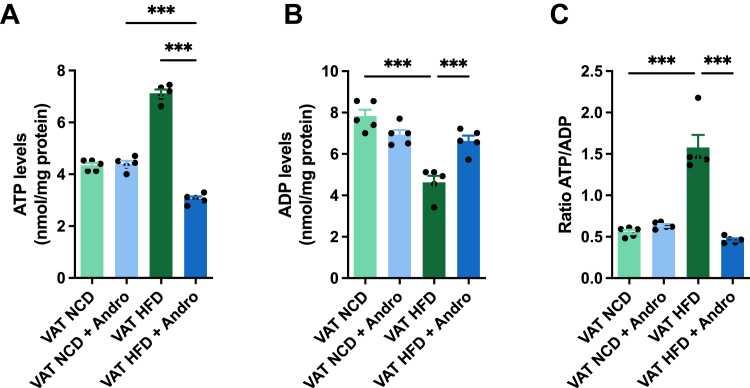
**Andro decreases ATP synthesis in VAT of HFD-fed mice.** VAT of mice fed with either NCD or HFD, treated in the presence or absence of Andro, was isolated and (*A*) ATP levels, (*B*) ADP levels, and (*C*) the ATP/ADP ratio was measured accordingly. One-way ANOVA followed by Bonferroni's *post hoc* test was performed. Data are represented as the mean values ± SD (n = 5 mice).

Next, to explore the impact of the observed glycolytic changes on ATP synthesis, we assessed ATP concentrations in VAT. Our findings indicated no alterations in ATP levels in the VAT among the NCD groups (Fig. 3*A*). However, there was a notable increase in ATP levels in the VAT of HFD-fed mice. Interestingly, the administration of Andro led to a substantial 50% reduction in ATP levels in the VAT of HFD-fed mice, reaching similar levels observed in the NCD group. Next, we evaluated ADP levels and the ATP/ADP ratio to determine whether Andro’s effect on ATP was attributed to enhanced release or production. In the NCD group, Andro treatment induced a slight, though non-significant reduction in VAT’s ADP levels compared to the control animals (Fig. 3*B*). However, when we compared the untreated NCD and HFD-fed animals, there was a significant decrease in the VAT’s ADP levels in the latter group. Interestingly, Andro treatment in HFD-fed animals increased the VAT’s ADP levels by approximately 40%, suggesting that Andro limited the synthesis of ATP in this group. As expected, no significant differences were observed in the ATP/ADP ratio among the NCD groups (Fig. 3*C*). However, the HFD resulted in a threefold increase in the VAT’s ATP/ADP ratio in untreated animals compared to the Andro-treated group.

### Andro decreases the activity of glucose metabolic regulators in VAT

**Figure 4:**
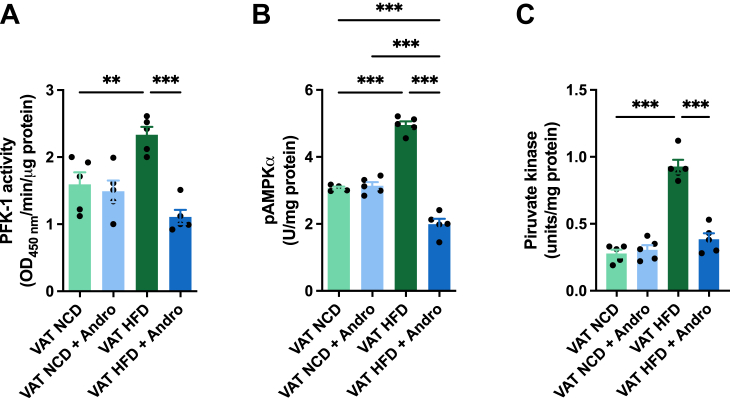
**Andro decreases the activity of glucose metabolic regulators in VAT.***A*, quantification of the activity of phosphofructokinase 1 (PFK-1). One-way ANOVA followed by Bonferroni's *post hoc* test was performed. *B*, quantification of the concentration of AMP-activated protein kinase (AMPK) phosphorylated at Thr172. One-way ANOVA followed by Bonferroni's *post hoc* test was performed. *C*, quantification of the activity of pyruvate kinase of VAT after indicated treatments. One-way ANOVA followed by Bonferroni's *post hoc* test was performed. Data are represented as the mean values ± SD (n = 5 mice).

To further investigate the impact of Andro on VAT, we also analyzed the activity of key metabolic regulators involved in glucose metabolism. We assessed the activity of phosphofructokinase 1 (PFK-1), AMP-activated protein kinase (AMPK), and pyruvate kinase. No significant differences in PFK-1, AMPK, and pyruvate kinase activities were observed when VAT was treated with Andro in NCD-fed mice compared to the control group (Fig. 4, *A*–*C*). However, in HFD-fed animals, a substantial increase in all enzymatic activities was noted. Andro treatment led to a significant reduction in PFK-1, AMPK, and pyruvate kinase activities in the VAT of HFD-fed animals (Fig. 4, *A*–*C*).

### Insulin reduces the effect of Andro on glucose uptake in HFD-fed animals

**Figure 5:**
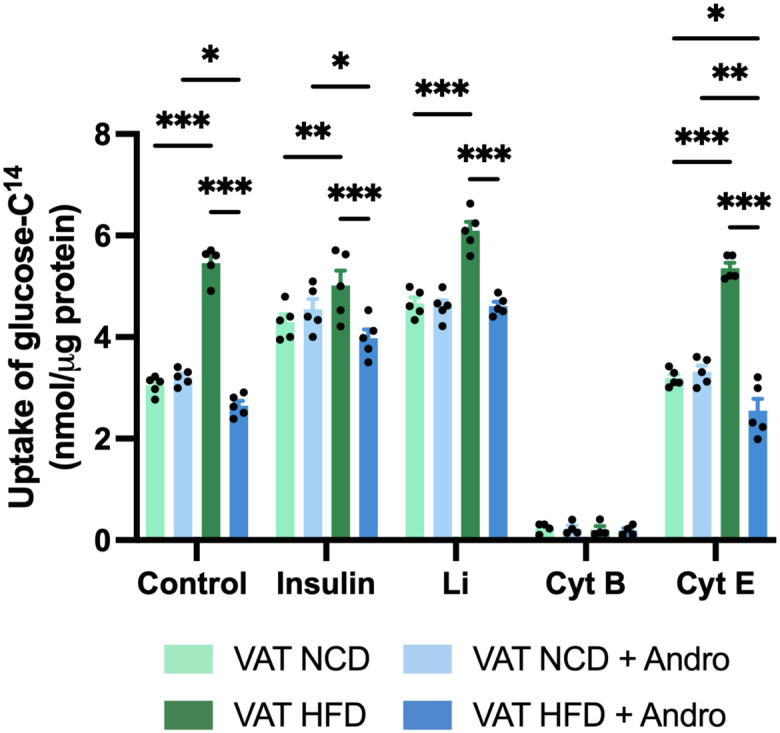
**Andro reduces glucose uptake in the VAT of HFD-fed mice in the presence of insulin and lithium.** VAT obtained from animals fed with either NCD or HFD was treated with insulin, lithium (Li), cytochalasin B (Cyt B), or cytochalasin E (Cyt E), and glucose uptake was measured. Two-way ANOVA followed by Bonferroni's *post hoc* test was performed. Data are represented as the mean values ± SD (n = 5 mice).

Next, we then monitored the uptake of glucose using various glucose uptake modulators or inhibitors such as lithium, insulin, cytochalasin (Cyt) B, or Cyt E. Given insulin’s role in regulating glucose uptake by promoting the glucose transporter 4 (Glut4) translocation to the cell membrane, we next explore the effect of insulin on glucose uptake (39). As expected, insulin increased glucose uptake in both NCD-fed mice groups as compared to control NCD groups with no apparent Andro effect (Fig. 5). Notably, although the uptake of glucose rose significantly in insulin-treated HFD-fed animals, compared to the insulin-treated NCD group, the extent of the effect was similar as in untreated control HFD-fed mice. Importantly, in the presence of insulin, Andro led to a significant 14% decrease in glucose uptake in the HFD-fed mice group compared to control HFD mice. Lithium has been previously demonstrated to act as a stimulator of cerebral glucose metabolism (40, 41, 42). Thus, we evaluated whether lithium could alter glucose uptake in the VAT of APP/PS1 mice. In accordance with previous results, glucose uptake in the VAT of HFD-fed mice increased by approximately 30% compared to the VAT of the NCD-fed control group. On the other hand, Andro treatment reduced the glucose uptake by approximately 24% compared to the control group of HFD-fed mice. Next, we treated VAT with two inhibitors of actin polymerization, namely Cyt B, a robust inhibitor of glucose transporter, and Cyt E, an analog of Cyt B that does not impede glucose transport. As expected, Cyt B strongly suppressed glucose uptake in all mouse groups and Cyt E resulted in similar glucose uptake levels as in the untreated control conditions.

### Andro regulates the mRNA expression of different metabolic genes in VAT

**Figure 6:**
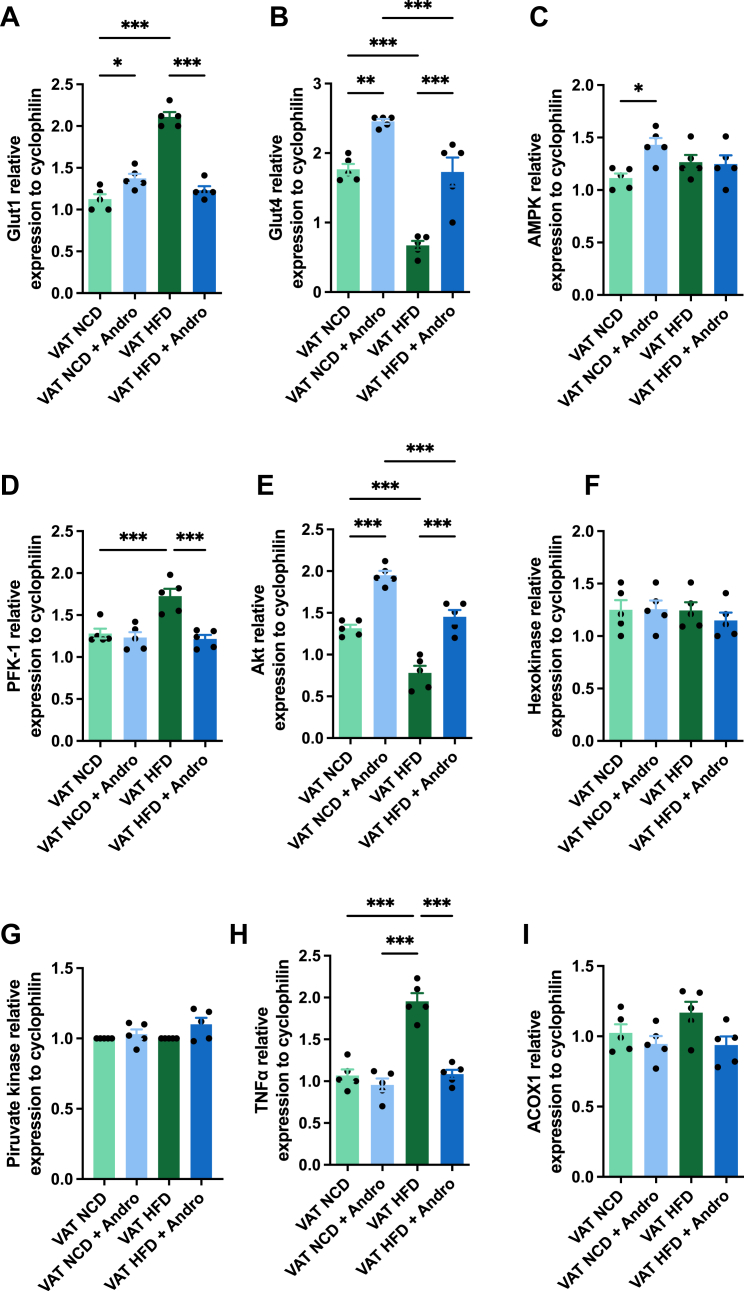
**Andro regulates the mRNA expression of different metabolic genes in VAT.** The VAT of mice subjected to NCD or HFD in the presence or absence of Andro was isolated and mRNA expression levels of (*A*) glucose transporter (GLUT) 1, (*B*) GLUT4, (*C*) AMP-activated protein kinase (AMPK), (*D*) phosphofructokinase 1 (PFK-1), (*E*) AKT, (*F*) hexokinase, (*G*) pyruvate kinase, (*H*) tumor necrosis factor (TNF)-α and (*I*) acyl-CoA oxidase 1 (ACOX1) were measured. One-way ANOVA followed by Bonferroni's *post hoc* test was performed. Data are represented as the mean values ± SD (n = 5 mice).

Previous studies have shown that Andro alters the expression of various metabolic genes in the brain (43). To comprehensively understand the impact of Andro on VAT, we also assessed the expression levels of several genes associated with glucose metabolic activity. First, we measured the expression changes of Glut1, crucial for basal glucose transport, and Glut4, the predominant isoform responsible for insulin-stimulated glucose uptake in adipocytes. Strikingly, Glut1 mRNA expression increased approximately 1-fold in the VAT of HFD-fed animals compared to the NCD control mice group (Fig. 6*A*). However, Andro significantly reduced Glut1 expression in the HFD group. Interestingly, we observed opposite results on Glut4 expression (Fig. 6*B*). Andro treatment in NCD-fed mice led to a ∼40% increase in Glut4 mRNA expression, while HFD-fed mice exhibited a significant ∼62% decrease compared to control animals. Notably, Andro treatment increased Glut4 expression by ∼2.6-fold compared to non-treated HFD-fed mice, reaching levels similar to those of control animals in NCD-fed mice.

Subsequently, we examined the effect of Andro on the expression of key metabolic regulators, such as AMPK, PFK-1, Akt, HK, and pyruvate kinase. A slight but significant upregulation of AMPK was observed after Andro treatment in the NCD-fed animals, compared to the untreated animals (Fig. 6*C*). However, the administration of HFD caused no changes in AMPK expression, independent of the presence of Andro. Next, we examined the mRNA expression of PFK-1 and observed no significant differences in animals on the NCD diet upon Andro treatment (Fig. 6*D*). However, mice on the HFD diet exhibited a notable upregulation of PFK-1, which was downregulated by approximately 30% after Andro treatment. Additionally, we measured the expression levels of Akt, an energy sensor that plays a central role in glucose metabolism and is known to be altered in obesity (44, 45, 46). Abnormal Akt signaling is also associated with insulin resistance, type 2 diabetes, metabolic syndrome, and glucose and lipid metabolism disorders (45, 46). Our findings showed an Andro-mediated increase in Akt expression in NCD-fed animals (Fig. 6*E*). However, animals on HFD showed a strong downregulation of Akt expression in comparison to the lean animals, an effect that was reversed upon Andro treatment. Finally, we analyzed the expression changes of both hexokinase and pyruvate kinase. We found no significant differences in the expression levels of either gene among the groups (Fig. 6, *F* and *G*).

In addition to these metabolic regulators, we also evaluated the mRNA expression of tumor necrosis factor alpha (TNF-α), a key pro-inflammatory cytokine associated with metabolic inflammation in adipose tissue (47) and acyl-CoA oxidase 1 (ACOX1), a peroxisomal enzyme involved in β-oxidation (48), to explore potential changes in inflammatory tone and fatty acid oxidation capacity in VAT. As shown in Figure 6*H*, HFD feeding led to a significant upregulation of TNF-α expression compared to the NCD group, indicating a heightened inflammatory state. Remarkably, Andro treatment significantly reduced TNF-α expression in the HFD group, suggesting an anti-inflammatory effect of the compound in VAT. In contrast, no significant differences in ACOX1 expression were observed among the experimental groups (Fig. 6*I*), suggesting that peroxisomal β-oxidation is not substantially modulated by Andrographolide under the conditions tested.

## Discussion

Obesity and an increase in VAT mass have been linked to cognitive impairment (27, 49, 50, 51, 52, 53, 54, 55). While the precise mechanism remains elusive, accumulating evidence suggests that it may be attributed to the alteration of the adipose tissue-brain axis, involving the dysregulation of adipocyte-derived signaling molecules (56, 57, 58, 59). Therefore, the use of pharmacological compounds capable of restoring adipocyte function holds great potential to alleviate AD symptoms (60, 61). In this study, we demonstrate that Andro, a natural anti-diabetic compound known to enhance glucose metabolism in the brain (62), successfully mitigated obesity-linked alterations in glucose metabolism in the VAT of APP-PS1 mice. These findings offer evidence supporting the potential therapeutic application of Andro in addressing metabolic dysregulations associated with obesity in AD.

An unexpected finding was the elevated basal glucose uptake levels observed in HFD animals, compared to those on the NCD. This observation aligns with previous studies indicating increased glucose uptake in adipocytes derived from mice with obesity and in patients with diabetes mellitus (63, 64, 65). Nevertheless, it remains controversial whether obesity alters glucose uptake since insulin resistance is strongly associated with reduced glucose uptake in insulin-responsive tissues (28). Indeed, some groups have shown that obesity also inhibits or does not alter glucose uptake in adipose tissue (66, 67). Interestingly, the increase in glucose uptake was also associated with the downregulation of Glut4 expression in HFD mice, whereas Glut1 expression was strongly upregulated. The insulin-regulated Glut4 is the most abundant transporter isoform in adipose tissue, and its expression is downregulated during obesity and type 2 diabetes (68, 69, 70). Earlier research has also demonstrated impaired translocation and altered distribution of Glut4 in the adipose tissue of mouse models with diabetes (71, 72, 73). Furthermore, the specific ablation of Glut4 in adipose tissue results in glucose intolerance, hyperinsulinemia, and insulin resistance (70). Conversely, mice overexpressing adipose Glut4 exhibit enhanced glucose tolerance (74). In contrast to Glut4, we observed a significant upregulation of Glut1 expression in HFD animals. An increase in Glut1 expression and protein levels has been previously reported in adipose tissue from obese mice (64, 75, 76). Interestingly, despite being less abundant than Glut4 in adipocytes, Glut1 not only responds to insulin but also to a variety of other stimuli, making this protein less susceptible to the effects of diabetes (77). Hence, the observed increase in glucose uptake may be caused by Glut1 upregulation or by other Glut members that facilitate glucose uptake in obesity, rather than Glut4 (64, 77). In addition to the alterations in glucose uptake observed in VAT, our data reveal systemic metabolic changes induced by HFD and partially reversed by Andro treatment. Notably, Glut1/4 the ability of Andro to counteract HFD-induced alterations in metabolic parameters supports a systemic improvement in glucose handling. These findings suggest that Andrographolide not only modulates VAT-specific metabolism but also exerts beneficial effects on whole-body glucose homeostasis in APP/PS1 mice with obesity, thereby contributing to an overall improvement in metabolic health. This interpretation is further supported by the improved glucose clearance observed in the GTT, as evidenced by the significantly reduced AUC in Andrographolide-treated HFD-fed animals compared to their untreated counterparts. These findings suggest that Andrographolide improves systemic glucose disposal capacity in the context of diet-induced metabolic dysfunction.

It is noteworthy that HFD stimulated glycolysis and reduced PPP, whereas Andro administration resulted in the opposite effect. Previous studies have indicated that hypoxia, a condition occurring in adipose tissue during obesity, not only increases glucose uptake but also upregulates the expression of various glycolysis-related genes, including Hexokinase and PFK-1 (78, 79). Additionally, exposure of adipocytes to hypoxic conditions raises Glut1 levels and reduces Glut4 expression, potentially compromising insulin sensitivity (75, 80). Further studies evaluating VAT hypoxia in APP-PS1 mice are needed to establish possible causal relationships between this condition and the expression of Glut1/4 and glycolysis-related molecules.

In several AD models, impaired glucose metabolism in the brain has been consistently reported (81, 82). Notably, reduced glucose utilization can be detected as early as 10 to 20 years before clinical symptoms appear (83, 84, 85). This hypometabolism has been associated with decreased glucose uptake and mitochondrial dysfunction, contributing to the progression of the disease (10, 36, 41, 86). During the early stages of AD, a compensatory upregulation of the PPP has been reported, likely aimed at counteracting oxidative stress. However, as the disease advances, this response becomes insufficient, leading to a simultaneous reduction in both oxidative phosphorylation and PPP activity, thereby exacerbating oxidative damage and neuronal dysfunction (87, 88, 89, 90). Importantly, most of the literature on altered glucose metabolism in AD has focused on the brain, with limited information on peripheral tissues such as VAT. The findings reported in our study, namely a decrease in glycolytic flux and a restoration of PPP-related activity in VAT from APP/PS1 mice treated with Andrographolide, suggest that metabolic dysregulation in AD extends beyond the central nervous system. These results emphasize the relevance of further exploring peripheral metabolic pathways in AD to better understand systemic alterations associated with the disease (91).

The activation of AMPK, an enzyme that acts as an energy sensor, promotes glucose uptake and the generation of cellular ATP, while reducing ATP consumption (92, 93, 94). Our findings suggest an increase in activated AMPK, high ATP levels, and an elevated ATP/ADP ratio in HFD-fed animals. This contrasts with previous studies showing HFD decreasing AMPK activity in adipose tissue from various rodent models and individuals with obesity (95, 96, 97). However, to our knowledge, there are no studies involving the function of AMPK in AD models. Importantly, an increase in AMPK activation in adipose tissue has been shown to protect mice obesity on a HFD by increasing energy expenditure (98). Conversely, the lack of AMPK activity in adipose tissue worsens the detrimental effects induced by a HFD and exacerbates insulin resistance (99). Thus, the observed increase in AMPK activity in the VAT of HFD-fed animals might arise as a compensatory mechanism to alleviate diet-induced obesity. It is worth noting that leptin and adiponectin, two adipokines known to be altered in obesity, activate AMPK in adipose tissue (100, 101). Therefore, it is possible that the observed activation of AMPK may be influenced by other pathways.

Additionally, ATP has been shown to play a significant proinflammatory role (102); the elevated ATP levels observed in the HFD-fed animals might also be linked to a systemic proinflammatory state. However, further studies are needed to determine whether these animals exhibit elevated inflammatory cytokines, as shown in AD-associated neuroinflammation (103).

Interestingly, we observed a reduction in glucose uptake in VAT together with reduced blood glucose concentration after Andro treatment. Previous studies conducted by our group and others have demonstrated that Andro promotes glucose uptake in a variety of tissues, including brain, pre- and mature adipocytes (31, 33, 36, 38, 62, 104). Andro has also been shown to lower plasma glucose in diabetic rats, potentially by enhancing glucose uptake in muscle cells (105). Thus, the observed decrease in blood glucose levels may be due to increased uptake by other organs. Interestingly, Andro's ability to enhance glucose uptake has mostly been observed in models unrelated to dementia. Therefore, the downregulation of glucose uptake by Andro in VAT from HFD-fed AD animals might be related to alterations caused by AD, such as systemic inflammation (106). This could, at least in part, explain the reduced glucose uptake by adipose tissue in response to Andro treatment.

Several studies have suggested that cognitive decline is exacerbated by insulin resistance, leading to a reduction of gray matter volume in several brain regions (107). Moreover, dysregulated insulin/insulin-like growth factor signaling and insulin resistance in the central nervous system have been associated with an elevated risk of dementia, including Alzheimer's disease (108, 109, 110, 111). On the other hand, the dysregulation of the Akt signaling pathway, which operates downstream of the insulin signaling, has been implicated in altered glucose metabolism, which is a key aspect of obesity-related metabolic dysfunction (112, 113). Notably, Akt plays a crucial role in promoting the translation and translocation of Glut4, thereby enhancing glucose uptake (114, 115). Interestingly, our study revealed a significant reduction in Akt expression, together with a downregulation of Glut4 in mice fed with an HFD. These findings align with existing evidence demonstrating diminished Akt levels in animals exposed to an HFD (116).

Consistent with the glucose-related effects observed in VAT, our findings also revealed that Andrographolide significantly reduced the expression of TNF-α in HFD-fed mice. This result supports the anti-inflammatory potential of Andrographolide in adipose tissue, in agreement with previous reports demonstrating its ability to suppress inflammatory mediators in metabolic and neurodegenerative contexts (117, 118). The marked upregulation of TNF-α in adipose tissue during obesity is a well-established marker of adipose tissue dysfunction and insulin resistance (119), and its attenuation by Andrographolide may contribute to the observed effects on glucose metabolic regulators in VAT. In contrast, ACOX1 expression remained unchanged among experimental groups, suggesting that Andrographolide’s metabolic actions in VAT are not mediated by modulation of peroxisomal lipid oxidation at the transcriptional level. Together, these findings indicate that the beneficial effects of Andrographolide on VAT glucose metabolism may be partially attributed to its anti-inflammatory actions, rather than to enhanced lipid catabolism; however, additional studies are necessary to confirm this hypothesis.

Additionally, the inclusion of hepatic enzyme measurements in the current study provides further insight into the systemic metabolic impact of Andrographolide. The absence of significant alterations in these markers across groups suggests that Andrographolide does not induce hepatotoxicity and may preserve liver function under high-fat diet conditions, further supporting its metabolic safety profile.

While our study represents the first exploration of Andro on VAT and its effects on AD, it comes with certain limitations. Although we demonstrated the restoration of adipocyte function after Andro treatment, we did not analyze its effect on AD. In earlier studies, both we and others have highlighted the positive implications of Andro in AD pathology (62, 120, 121). Moreover, a recent study from our lab focused on the effect of adipokines in HFD-fed APP/PS1 mice. Interestingly, we observed alterations in Aβ pathology, cognitive functions, and glucose metabolism, among others (122). An additional limitation is the lack of a detailed mechanistic understanding of Andro in the VAT. In the brain, we have shown that Andro activates the Wnt signaling pathway by inhibiting glycogen synthase kinase-3β (GSK-3β) (123, 124, 125). However, its role in the VAT has yet to be elucidated. Thus, future investigations should delve deeper into the molecular mechanisms underlying the effect of Andro, considering its potential in mitigating AD pathology.

Previous studies have shown that Andrographolide can modulate key metabolic signaling pathways in wild-type models, including the activation of Wnt/β-catenin signaling in C57BL/6J mice (123) and the stimulation of glucose uptake and AMPK activation in primary hippocampal neurons from neonatal rats (36). However, further research is needed to assess its impact on systemic metabolic health under physiological conditions.

Although body weight was not significantly reduced by Andrographolide treatment, our findings suggest that the compound improves glucose metabolism in VAT through tissue-specific actions, independent of overall body weight changes. This interpretation aligns with recent clinical studies showing that metabolic benefits can occur without weight loss, even under stable body weight conditions (126). These observations reinforce the notion that adipose tissue functionality, rather than mass alone, may be a critical target in metabolic interventions.

A limitation of our study is the absence of a wild-type (WT) HFD-fed control group, which restricts our ability to fully discriminate between metabolic alterations driven by the APP/PS1 genotype and those induced solely by high-fat diet exposure. Nonetheless, evidence from previous studies in WT animals by Tapia-Rojas *et al.* (127) demonstrated that Andrographolide exerts neuroprotective effects in WT mice through inhibition of GSK-3β and activation of the canonical Wnt/β-catenin pathway, a signaling cascade that is also implicated in adipose tissue homeostasis and inflammation. These findings suggest that Andrographolide may modulate metabolic function in a genotype-independent manner, although further studies directly comparing WT and transgenic models are warranted to confirm this hypothesis. In addition, the present study was conducted exclusively in male mice at 4 months of age. While this design minimizes hormonal variability and corresponds to young adulthood, a commonly used time point in metabolic studies, it may limit the generalizability of our findings. Future studies should therefore incorporate both sexes and different age groups to assess the broader translational potential of Andrographolide-based interventions.

In summary, exploring the impact of Andro on adipose tissue metabolism yields promising insights into its potential applications, particularly in the context of AD. The observed improvements in glucose metabolism within VAT suggest a multifaceted impact of Andro on cellular responses. Considering the established connection between metabolic dysregulation, obesity, and the risk of developing AD, interventions that target adipose tissue functionality hold substantial promise. Thus, the dual role of Andro in mitigating both metabolic and neurodegenerative aspects of AD positions it as a valuable pharmacological candidate for further research.

## Experimental procedures

### Animals and ethical standards

Male APP/PS1 (RRID: MMRRC_34829-JAX) were used in this study. APP/PS1 animals co-express the Swedish (K594M/N595L) mutation of a chimeric mouse/human APP (Mo/HuAPP695swe) together with the human exon-9-deleted variant of PS1 (128, 129). We divided the animals into four groups with five to six animals in each group (Fig. 1*A*). In the first and second groups, 4-month-old animals were fed with NCD (with 10% energy from fat, #7024, TestDiet) and received intraperitoneal injections of vehicle (saline solution 0.9% NaCl) or Andro (2 mg/kg, #365645, Sigma-Aldrich), respectively (123, 130). The third and fourth groups were fed an HFD, with 45% energy from fat, #58G8, (TestDiet), and received intraperitoneal injections of saline or Andro (2 mg/kg), respectively. Injections of either Andro or saline solution as a vehicle were carried out three times per week for 16 weeks. After treatments, animals were euthanized by decapitation following asphyxiation by isoflurane. After euthanasia, the VAT was removed from the regions around de branches of the superior and inferior mesenteric arteries. All the experiments were normalized by the protein expression of VAT samples. Animals were maintained at the Animal House Facility of the Pontificia Universidad Católica de Chile under a sanitary barrier in ventilated racks and in closed colonies; no sample calculation was performed. We used simple randomization to allocate mice to different cages. Experimental procedures were approved by the Bioethical and Biosafety Committee of the Faculty of Biological Sciences of the Pontificia Universidad Católica de Chile with the ethical approval CBB-158/2014. The inclusion/exclusion criteria for this study were the health of the animals after treatment; in this study, no animals were excluded. Measurements of animal weight, food, and liquid intake were obtained once per week during the treatment.

### Biochemical analysis

After treatments, glucose, cholesterol, insulin, triglycerides, alkaline phosphatase, and aspartate aminotransferase (AST) were measured from blood samples obtained from 5 to 6 mice per group. Intracardiac blood was collected before decapitation, after 6 h of fasting. Serum samples were obtained and stored at −20 °C for later analysis. Glucose levels were measured according to the hexokinase/G-6-PDH method using Architect Analyzer (Abbott Laboratories). Insulin levels were measured *via* chemiluminescence (Beckman Coulter) and cholesterol levels were enzymatically assessed using an Architect c8000 analyzer. HOMA-IR, an insulin resistance index, was calculated using the following formula: HOMA-IR = fasting glucose (mmol/L) × fasting insulin (mU/ml)/22.5. Triglycerides were assessed enzymatically using the Architect c8000 analyzer (Abbott Laboratories). Alkaline phosphatase activity was measured in the stool supernatant using an automatic biochemistry analyzer. For this, 20 μl of supernatant was added to 1 ml of assay buffer containing p-nitrophenyl phosphate (pNPP) as a substrate and incubated for 1 min at 37 °C. The resulting Alkaline phosphatase activity was then quantified by the analyzer, which was previously calibrated with Alkaline phosphatase standards (131). AST was measured using an automatic blood chemical analyzer (Hitachi) (132). For the GTT, after 30 days of special or standard diet, animals were fasted for 8 h and then received an injection of glucose (1 g/kg b.w., i.p.). Blood glucose was monitored at 20, 30, 60 and 120 min using a glucometer (Accu-Check) on samples collected from the tip of the tail vein. The AUC analysis was calculated using GraphPad software (133).

### Glucose uptake analysis

The VAT was prepared according to standard procedures. Briefly, VAT samples were washed with washing buffer (15 mM HEPES [#H3375, Sigma Millipore], 135 mM NaCl [#s3014], 5 mM KCl [#P5405], 1.8 mM CaCl_2_ [#C1016], and 0.8 mM MgCl_2_ [#208337], all from Sigma-Aldrich (St Louis, MO, USA), supplemented with 0.5 mM glucose. The tissue was incubated with 1-1.2mCi D-[1-^14^C] glucose (#NEC043, PerkinElmer, MA, USA) at a final specific activity of 1 to 3 disintegrations/min/pmol (∼1 mCi/mmol), for indicated periods. Glucose uptake was arrested by washing the VAT with ice-cold PBS supplemented with 1 mM HgCl_2_ [#203777, Sigma- Aldrich]. The incorporated radioactivity was then quantified by liquid scintillation counting, as previously described by us (36). All data were normalized to VAT protein content. When indicated, the VAT was incubated for 30 min with either insulin, Li_2_CO_3_ [Li, 10 mM, #554-13-2], Cyt B [20 μM, #C6762], or Cyt E [20 μM, #C2149], reagents from Sigma-Aldrich.

### Determination of the glycolytic rate

Glycolytic rates were determined as previously described (122). Briefly, VAT was placed in tubes containing 5 mM glucose and then washed twice in Krebs–Henseleit solution (11 mM Na_2_HPO_4_, 122 mM NaCl, 3.1 mM KCl, 0.4 mM KH_2_PO_4_, 1.2 mM MgSO_4_, and 1.3 mM CaCl_2_, pH 7.4) containing the appropriate concentration of glucose. After equilibration in 0.5 ml of Hank’s balanced salt solution/glucose [#14025076, Thermo Fisher, MA, USA] at 37 °C for 30 min, 0.5 ml of Hank’s balanced salt solution containing various concentrations of D-[1-^14^C] glucose (#NEC043, PerkinElmer, MA, USA) was added, with a final specific activity of 1 to 3 disintegrations/min/pmol (∼1 mCi/mmol). A vapor-phase equilibration step was performed by mixing the samples with water, and then the mixture was incubated at 45 °C for 48 h. The ^3^H_2_O content in the scintillation mixture was determined by counting over 5 min. We expressed the results in mg of protein per tissue.

### Hexokinase activity

To measure the hexokinase activity, the VAT was washed with PBS, treated with trypsin/EDTA, and centrifuged at 500*g* for 5 min at 4 °C. Lysates were then resuspended in isolation medium (250 mM sucrose, 20 mM HEPES, 10 mM KCl, 1.5 mM MgCl2, 1 mM EDTA, 1 mM DTT, 2 mg/ml aprotinin [#A1153], 1 mg/ml pepstatin A [#77170], and 2 mg/ml leupeptin [#L8511], reagents from Sigma-Aldrich (St Louis, MO, USA), at a 1:3 dilution, sonicated at 4 °C, and then centrifuged at 1500*g* for 5 min at 4 °C. The supernatant fraction was mixed with the reaction medium (25 mM Tris-HCl, 1 mM DTT, 0.5 mM NADP/Na^+^, 2 mM MgCl_2_, 1 mM ATP, 2 U/ml G6PDH, and 10 mM glucose), and incubated at 37 °C for 30 min. The reaction was stopped by the addition of 10% trichloroacetic acid, TCA, [#T6399, Sigma- Aldrich ], and the generation of NADPH was measured at 340 nm (10).

### Pentose phosphate pathway (PPP) measurement

Glucose oxidation *via* the PPP was measured based on the difference in ^14^CO_2_ production from D-[1-^14^C] glucose and D-[6-^14^C] glucose, as previously described (122). To this end, VAT tissue was washed with ice-cold PBS and kept in O_2_-saturated Krebs Henseleit buffer. Samples were then placed in Erlenmeyer flasks with another 0.5 ml of the Krebs Henseleit solution containing 0.5 mCi D-[1-^14^C] glucose (#NEC043, PerkinElmer, MA, USA) or 2 mCi D-[6-^14^C] glucose (#NEC045, PerkinElmer) and 5.5 mM D-glucose (final concentration). The Erlenmeyer flasks were equipped with a central well containing an Eppendorf tube with 500 μl of benzethonium hydroxide. The flasks were flushed with O_2_ for 20 s, sealed with rubber caps, and incubated for 60 min in a 37 °C water bath with shaking. The incubations were stopped by the injection of 0.2 ml of 1.75 M HClO_4_ into the main well, and shaking was continued for another 20 min to facilitate the trapping of ^14^CO_2_ by benzethonium hydroxide. Radioactivity was assayed by liquid scintillation spectrometry.

### Determination of G6PDH activity

VAT tissue samples were washed with PBS, collected by trypsinization (0.25% trypsin- 0.2% EDTA (w/v)), and pelleted. The tissue samples were then resuspended in isolation medium (250 mM sucrose, 20 mM HEPES, 10 mM KCl, 1.5 mM MgCl_2_, 1 mM EDTA, 1 mM DTT, 2 mg/ml aprotinin, 1 mg/ml pepstatin A, and 2 mg/ml leupeptin) at a 1:3 dilution, sonicated at 4 °C, and centrifuged for 5 min at 1500*g* at 4 °C. Subsequently, the supernatant was further separated by centrifugation at 13,000*g* for 30 min at 4 °C. Finally, the G6PDH activity of the supernatant was quantified in a reaction buffer containing 1 mM ATP and 10 mM glucose-6-phosphate (G6P) for 30 min at 37 °C. The reaction was stopped by the addition of 10% TCA. The generation of NADPH was measured at 340 nm, as described previously (134).

### Quantification of ADP and ATP levels

ATP and ADP levels were measured using an ATP determination kit [#A22066, Invitrogen/Molecular Probes] or an ADP assay Kit [#ab83359, Abcam, Cambridge, United Kingdom], respectively. The ADP/ATP ratio was measured accordingly (122).

### AMPK, phosphofructokinase-1 (PFK-1), and pyruvate kinase Activity

AMPK activity was measured by ELISA according to the manufacturer’s instructions [#KHO0651, ThermoFisher Scientific]. PFK-1 activity was measured using a Colorimetric Assay Kit [#K776, BioVision] (135). Pyruvate kinase activity was measured using a pyruvate kinase assay kit [#ab83432, Abcam] (136, 137).

### Quantitative real-time PCR (qRT-PCR)

RNA was isolated from VAT and reverse transcribed into cDNA [#18091050, Invitrogen]. Quantitative real-time RT–PCR (qRT–PCR) was conducted using SYBR master mix [#4368577, ThermoFisher Scientific], with the program recommended by the manufacturer and as published previously (138). As a reference, we used the housekeeping gene cyclophilin, and the relative Ct values of each gene were calculated using the delta Ct, in comparison with the control gene. Duplicated control reactions for every sample without reverse transcription were included to ensure that PCR products were not due to the amplification of contaminated genomic DNA. The sets of primers (IDT Integrated DNA Technologies, IA, USA) used are detailed in Table S1.

### Statistical analysis

All Statistics were performed using the software Prism 9 (GraphPad). The results are expressed as means ± standard deviation (SD). Data were analyzed by one or two-way analysis of variance (ANOVA), followed by Bonferroni’s *post hoc* test; ∗*p* < 0.05, ∗∗*p* < 0.01, and ∗∗∗*p* < 0.001 were considered significant. To test the presence of outliers, we used the Prism software; however, no outliers were detected.

## Data availability

Data will be made available on request.

## Supporting information

This article contains supporting information.

## Conflict of interest

The authors declare that they do not have any conflicts of interest with the content of this article.
