# Supplementary material for: Andrographolide modulates glucose metabolism in visceral adipose tissue in an Alzheimer's disease obese mouse model
Source: J Biol Chem. 2025 Aug 16;301(10):110607. doi: 10.1016/j.jbc.2025.110607 (PMC12481917; doi:10.1016/j.jbc.2025.110607)
Supplement: Table S1 [file mmc1.docx]

| **Table S1:** List of primers used in the study. | |  |  |  |
| --- | --- | --- | --- | --- |
|  |  |  |  |  |
| **Gene** | **Forward** | **Reverse** |  |  |
| *Cyclophilin* | 5’-TGGAGATGAATCTGTAGGAGGAG-3’ | 5’-TACCACATCCATGCCCTCTAGAA-3 | | |
| *Glut1* | 5’-ATGGATCCCAGCAGCAAGAAG-3’ | 5’-AGAGACCAAAGCGTGGTGAG-3’ | | |
| *Glut4* | 5’-CGGCTCTGACGATGGGGAA-3’ | 5’-TTGTGGGATGGAATCCGGTCCCGATA-3’ | | |
| *Hexokinase* | 5′-GGATGGGAACTCTCCCCTG-3′ | 5′-GCATACGTGCTGGACCGATA-3′ | | |
| *Phosphofructokinase-1 (PFK-1)* | 5′-AGGGCCTTGTCATCATTGGG-3′ | 5′-ACTGCTTCCTGCCTTCCATC-3′ | | |
| *Akt* | 5'-TCACGTGAGCCCTTCTCCTA-3′ | 5'-CTCCCACCCACTAACAAGGC-3' | | |
| *AMPK (alpha subunit)* | 5'-GTGAAGATCGGCCACTACATCC-3' | 5'-GGCTTTCCTTTTCGTCCAACC-3' | | |
| *Pyruvate kinase* | 5'-CAGCATCATTGCCACCATCG-3' | 5'-GACTCCAGTGCGTATCTCGG-3' | | |
| *Tumor necrosis factor-α (TNF-α)* | 5'- TGATCGGTCCCCAAAGGGAT -3' | 5'- TGTCTTTGAGATCCATGCCGT -3' | | |
| *Acyl-CoA oxidase 1 (ACOX1)* | 5'- CGTAGCCAGCGTTATGAGGT -3' | 5'- TCCAGGCAGGCATGAAGAAG -3' | | |
